# Ultrasound-Guided Pericapsular Nerve Group Block for Hip Surgery: A Randomized Controlled Trial Study Comparing Ropivacaine and Ropivacaine With Dexamethasone

**DOI:** 10.7759/cureus.34261

**Published:** 2023-01-27

**Authors:** Aswin Balasubramaniam, Suresh Kumar Naggaih, Sumanth Tarigonda, Ravi Madhusudhana

**Affiliations:** 1 Anaesthesiology, Sri Devaraj Urs Medical College, Kolar, IND

**Keywords:** analgesia, dexamethasone, hip surgery, peng, ropivacaine, vas

## Abstract

Background: Patients with hip fractures will be experiencing excruciating pain, which would prevent the ideal positioning of the patient for the neuraxial blockade. There is growing interest in using regional nerve blocks for pain in the elderly associated with a fractured hip. Pericapsular Nerve Group (PENG) block is gaining popularity as a provider of adequate analgesia in patients suffering from a hip fracture. The present study aimed to assess the effectiveness of PENG block using ropivacaine alone or ropivacaine with dexamethasone in reducing pain scores during patient positioning for the central neuraxial blockade and to compare the duration of postoperative analgesia.

Materials and methods: This randomized double-blinded study was conducted on patients posted for hip surgery under spinal anesthesia at a tertiary care referral hospital between January 2021 and May 2022. Twenty-eight patients (14 in each group) were randomly allocated to receive either group A (20 ml of 0.5% ropivacaine for PENG block) or Group B (20ml of 0.5% ropivacaine with 8mg Dexamethasone for PENG block) before patient positioning for subarachnoid block. Intra-operative hemodynamic variables, pain scores on a visual analog scale (VAS), at rest and with movement, before block, at the time of positioning for spinal anesthesia, time for first rescue analgesic request, and the total dosage of rescue analgesia in the first 24 hours after PENG block were measured.

Results: Pain scores at rest and with movement at baseline and at the time of positioning for spinal anesthesia were significant within the groups (p< 0.01). The time for the first rescue analgesic requirement was significantly longer in group B (445.0 ±17.4 minutes) than in group A (388.9±19.0 minutes) (p<0.05). The mean average number of doses of rescue analgesia (Tramadol in milligrams) was significantly lower in group B (190 ± 60) than in group A patients (250 ± 70) (p<0.05).

Conclusion: The present study documented the effectiveness of PENG for patient positioning during the neuraxial blockade. Further, the addition of dexamethasone as an adjunct to ropivacaine yields a significantly longer duration of postoperative analgesia with a lower postoperative analgesic requirement.

## Introduction

Hip fractures are common orthopedic problems associated with significant morbidity and mortality. As a definitive treatment, most patients with fractured hips would undergo early reduction and surgical fixation [1]. Central neuraxial blockade is the most common anesthetic technique for hip surgical procedures. Patients with hip fracture experience excruciating pain, which would prevent the ideal positioning of the patient for the neuraxial blockade [2]. There is growing interest in using regional nerve blocks for pain reduction in the elderly with fractured hips. A recent Cochrane review supports the usage of the regional blockade in reducing pain on movement within 30 min of block placement [3].

Femoral nerve (FN) block, Fascia Iliaca Block (FIB), and 3-in-1 FN block were formerly used to treat hip fractures. These blocks provided perioperative analgesia and reduced the patients' postoperative opioid requirement [4]. The femoral nerve, obturator nerve, and supplementary obturator nerve branches innervate the anterior hip capsule. Literature suggests that the obturator nerve is not covered in these blocks. Available evidence suggests that the PENG block has shown some promise in a small case of patients undergoing hip surgery, although further studies comparing this with FICB are required [5]. The primary objectives were a) to assess the effectiveness of PENG block using ropivacaine alone or ropivacaine with dexamethasone as an additive in reducing the VAS scores while positioning the patients for a neuraxial blockade and b) to compare the duration and quality of postoperative analgesia.

## Materials and methods

This parallel-designed, randomized, and double-blinded study was conducted at a tertiary care referral hospital between January 2021 and May 2022 after obtaining prior institutional ethical committee approval (No DMC/KLR/IEC/103/2021-22). Written informed consent was obtained from all the enrolled study participants. Study participants, observers, and data analysts were blinded to the intervention carried out.

Sample size

Based on the mean duration of difference in analgesia as reported in a study comparing 0.5% ropivacaine and 0.5% ropivacaine with magnesium sulfate in supraclavicular brachial plexus block for forelimb and hand surgeries [6], a total sample size of 28 was calculated, with 14 patients in each group, as the number of participants necessary to detect a difference of 30 minutes of analgesia duration with 80% power and alpha error of 5%, considering an average variance estimate of (28.5).

**Figure 1:**
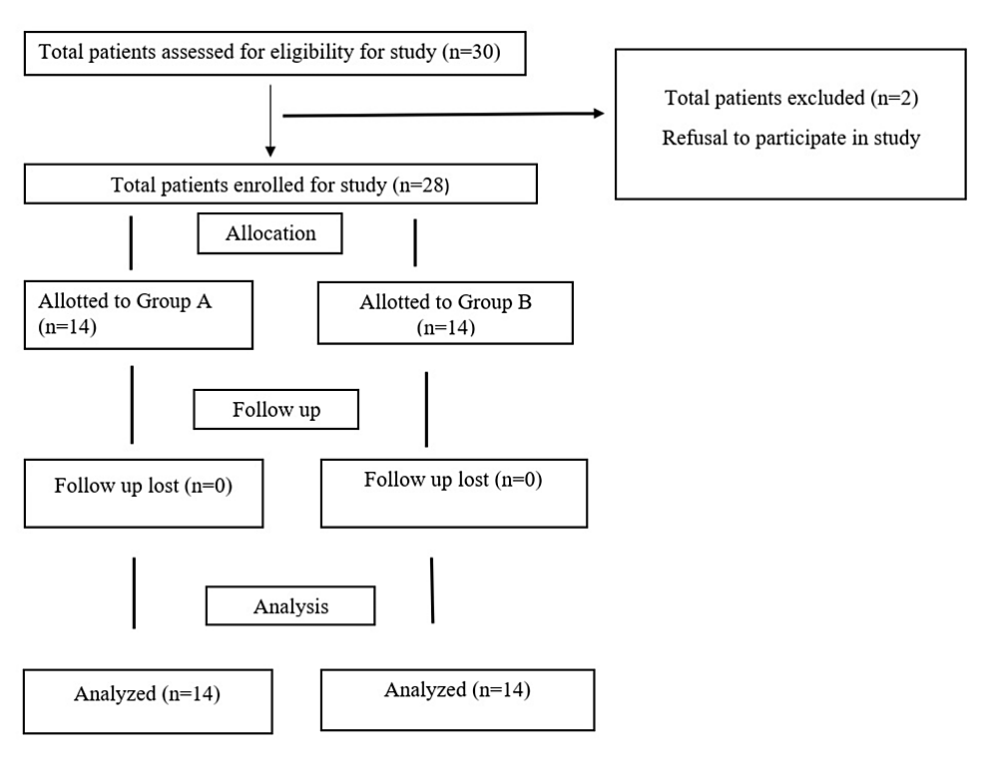
Consort flow diagram Group A: Patients who received a PENG block with 20ml of 0.5% ropivacaine Group B: Patients who received PENG block with 20ml of 0.5% ropivacaine and 8 mg dexamethasone

Patients older than 18 years with American Society of Anaesthesiologists (ASA) physical status 1-3 posted for elective surgery for hip fracture under spinal anesthesia were included in the study. Patients who refused to participate in the study, those with allergies to local anesthetics, deranged coagulation profile, infection at the block site, neurological deficits like paraplegia and paresis of the lower limb, and motor power less than 5/5 were excluded from the study. Study participants who satisfied the inclusion criteria were randomized using a computer-generated random number program (Figure 1).

Routine investigations, along with the coagulation profile, were checked on the day of surgery. Age, sex, ideal body weight, and ASA grading were recorded. Heart rate, non-invasive blood pressure (NIBP), and oxygen saturation (SpO2) were monitored throughout the procedure. Baseline VAS before the block was recorded. Patients were asked to rate the severity of pain using the VAS score at rest and with movement, which is noted as T0. Twenty minutes after performing the PENG block, the VAS score measured during the patient positioning for spinal anesthesia was recorded as TP. Patients were administered subarachnoid block in a sitting position at L3-L4 space with 0.5% of bupivacaine heavy 3ml with 25 micrograms of Fentanyl, using a 25G Quincke spinal needle. Other parameters observed were the duration of surgery, mean arterial pressure (MAP) less than 70 mm of hg was considered as hypotension after subarachnoid block (SAB), and it is treated either with fluid bolus or vasopressor. Time of first rescue analgesic (defined as the duration from the block placement till either patient requested an analgesic or VAS≥ 5), postoperative pain scores on VAS measured at 6, 12, and 24 hours at rest and also with movements and total consumption of rescue analgesia (tramadol) in 24 hours.

Description of PENG block

Philips Innosight (Philips Ultrasound Inc. USA) linear transducer probe (4-12 MHz) was placed at the level of the anterior superior iliac spine (ASIS) parallel to the inguinal crease and slide caudally in a gradual movement until the anterior inferior iliac spine (AIIS) was seen. The probe was rotated slightly medially until a continuous hyperechoic shadow of the upper pubic ramus was visible. The psoas muscle with a prominent tendon above the pubic ramus was visualized. The target for injection was identified in the plane between these two structures. The pubic ramus should be aligned in the center of the image to target it just inside the AIIS. 23G Quincke’s needle was inserted using the “In Plane” technique under vision to get the tip of the needle at the target site, i.e., between the iliopubic eminence and iliopsoas muscle.

Statistical analysis

Data were gathered, coded, and added to a Microsoft Excel (Redmond, USA) database. Qualitative measures like gender and all the quantitative measurements were reported by mean and standard deviation, confidence interval, measures of physical condition, and CI. To evaluate the data, independent sample t-tests, Mann-Whitney U-tests, and chi-square tests/exact Fisher's tests were used accordingly. Statistics were considered significant if the p-value was less than 0.05.

## Results

In the present study, a total of 28 patients were included after obtaining informed consent and divided into two groups (Group A-PENG block with 20 ml of 0.5% ropivacaine & Group B- PENG block with 20ml of 0.5% ropivacaine with 8mg dexamethasone) randomly, and all of them completed the study.

The mean age of group A was 54.8 ± 15.2, and that of group B was 47.2 ± 22.0; the difference between the two groups was statistically insignificant (p-value 0.654). Gender-wise, a male preponderance was noted, with 71.4% male and 28.6% female patients. The difference in the weight between the groups was statistically not significant (p-value 0.264) (Table 1).

**Table 1:** Comparison of the mean age and weight between the groups

Variables	Group A (n=14)		Group B (n=14)		p-value
	Mean	SD	Mean	SD	
Age in years	54.8	14.2	47.2	22.0	0.654
Weight in kilograms	65.9	7.9	63.5	13.1	0.264

**Figure 2:**
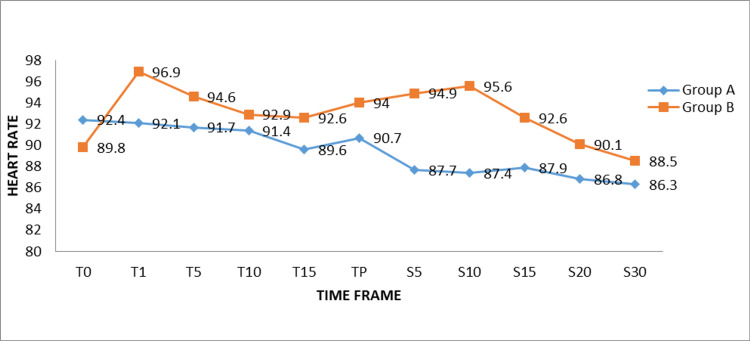
Comparison of mean heart rate between the groups at various time intervals PENG: Pericapsular nerve group, SAB: Subarachnoid block ,T0: Baseline, T1: At the time of PENG block, T5: 5 minutes after PENG block, T10: 10 minutes after PENG block, T15: 15 minutes after PENG block, TP: At the time of positioning patient for SAB, S5: 5 minutes after SAB, S10: 10 minutes after SAB, S15: 15 minutes after SAB, S20: 20 minutes after SAB, S30: 30 minutes after SAB

There was no discernible change in the research participants’ heart rates in the groups at different intervals (Figure 2).

**Figure 3:**
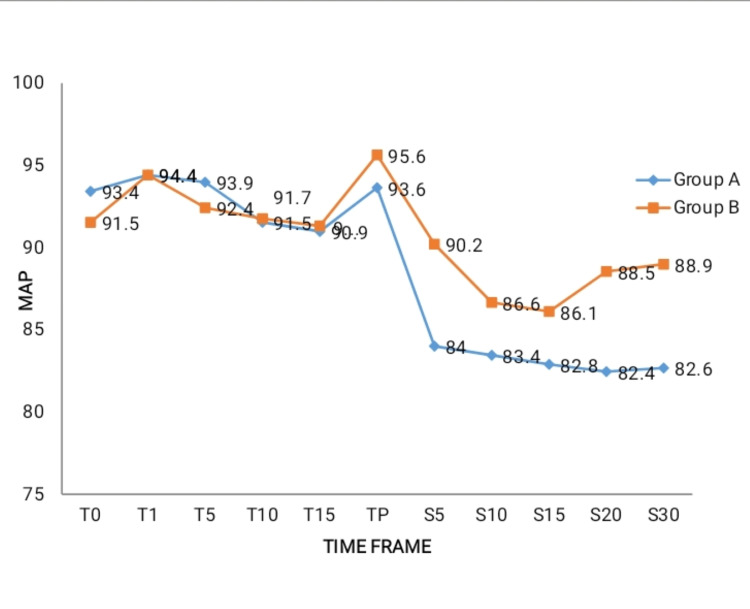
Comparison of mean arterial pressure between the groups at various intervals PENG: Pericapsular nerve group, SAB: Subarachnoid block, MAP: Mean arterial pressure, T0: Baseline, T1: At the time of PENG block, T5: 5 minutes after PENG block, T10: 10 minutes after PENG block, T15: 15 minutes after PENG block, TP: At the time of positioning patient for SAB, S5: 5 minutes after SAB, S10: 10 minutes after SAB, S15: 15 minutes after SAB, S20: 20 minutes after SAB, S30: 30 minutes after SAB

There was no discernible change in the research participants’ mean arterial pressure in both groups at different intervals (Figure 3).

There is a significantly lower VAS at rest during the time of positioning of the patient for SAB (TP) (group A 3.9 ± 1.2 and group B 3.2 ± 1.3) from baseline (T0) VAS at rest (group A 7.7 ± 1.6 and group B 7.4 ± 0.7) (p-value < 0.01). Similarly, there is a significantly lower VAS at movement during the time of positioning of the patient for SAB (TP) (group A 5.3 ± 1.1 and group B 5.0 ± 1.4) from baseline (T0) VAS at rest (group A 9.1± 1.3 and group B 9.4 ± 0.7) (p-value < 0.01). This reduction in VAS indicates the effectiveness of the PENG block for positioning patients for the neuraxial blockade (Table 2).

**Table 2:** Comparison of mean VAS at rest and movement within the groups VAS: Visual analog scale p-value < 0.05 - significant (paired t-test) * p-value - 0.01 - strong significance

VAS AT REST	Group A			Group B	
	Mean	SD		Mean	SD
Baseline (T0)	7.7	1.6		7.4	0.7
At the time of Positioning for SAB (TP)	3.9	1.2		3.2	1.3
p-value	0.01*			0.01*	
VAS WITH MOVEMENT	Group A			Group B	
	Mean		SD	Mean	SD
Baseline (T0)	9.1		1.3	9.4	0.7
At the time of Positioning for SAB (TP)	5.3		1.1	5.0	1.4
p-value	0.01*			0.01*	

In our study, the mean surgical duration was 86 ± 26 minutes. None of the patients had inadequate spinal anesthesia, and also complications/failure of PENG block was not seen. When group B patients were compared to those in group A, the period for the first rescue analgesic required (group B 445.0 ± 17.4 and group A 388.9 ± 19.0) was much longer (p<0.05). Similarly, group B patients had a much lower mean tramadol dose requirement (190 ± 60) than group A patients (250 ± 70). (p<0.05) (Table 3).

**Table 3:** Mean duration of the first rescue analgesic required and the dose of tramadol between the groups p-value < 0.05 - significant * p-value - 0.025 - strong significance ** p-value - 0.001 - very strong significance

	Group A		Group B		p-value
	Mean	SD	Mean	SD	
Time of first rescue analgesic in minutes	388.9	19.0	445.0	17.4	0.001**
Tramadol (in milligrams)	250	70	190	60	0.025*

## Discussion

Hip fracture patients feel excruciating pain, which would prevent the ideal positioning of the patient for the neuraxial blockade. In the past, Femoral Nerve (FN) block, Fascia Iliaca Block (FIB), and 3-in-1 block were used for hip fractures. These blocks not only provide perioperative analgesia but also reduce the opioid requirement in such patients. Branches from the femoral nerve, obturator nerve, and accessory obturator nerve innervate the anterior hip capsule. Current evidence suggests that an ultrasound-guided PENG block is more effective in blocking nerves than the blocks mentioned above.

The PENG block is a unique localized analgesic approach to lessen pain following total hip arthroplasties (THA) while preserving motor function, as L Girón Arango and colleagues initially suggested. The local anesthetic agent was deposited in the fascial plane, separating the superior pubic ramus and psoas muscle [7].

In patients undergoing hip fracture surgery, a PENG block administered before SAB gives better analgesia for appropriate positioning during the central neuraxial block. In addition, Ropivacaine, a long-acting local anesthetic, reduces CNS and cardiac toxicity and has less propensity for motor blockade than bupivacaine [8].

Studies have shown that adding dexamethasone to local anesthetic would significantly prolong postoperative analgesia [9]. The current study sought to determine the efficacy of PENG. Block using local anesthesia agent ropivacaine alone, compare with ropivacaine and dexamethasone with the help of pain score for patient positioning during the neuraxial blockade, and compare postoperative analgesia duration.

The mean age among participants was 51±18.6yrs, with a minimum age of 21 and maximum age of 85. A male preponderance was noted, with 71.4% male subjects and 28.6% female subjects. A comparison of mean age and weight reveals no discernible difference between the two groups.

There was no perceptible difference in either the heart rate or mean atrial pressure among the groups at various intervals. From the baseline, both groups achieved a significant reduction in VAS (at rest and with movements) during patient positioning during SAB after the PENG block (p<0.01%).

Huda AU et al. showed similar results regarding opioid consumption within the first 24 hours and duration of analgesia. They concluded that PENG block for hip surgery patients is associated with a substantial decrease in opioid intake (0.54 mg) in the first 24 hours following surgery (p=0.05) and increased duration of analgesia in the postoperative period [10]. Based on our study, the time for the first rescue analgesic requirement was substantially more in group B (445.0 minutes) than in group A (388.9 minutes) (p<0.05). Similarly, the mean dose of tramadol was comparatively less for group B than for group A patients (p<0.05).

G. Pascarella and colleagues studied the impact of PENG block on patients who had total hip replacement surgery [11]. They found significantly less post-operative pain scores in patients who received PENG block. Further, patients with PENG block had a considerable reduction in opioid intake, a better range of hip mobility, and a shorter ambulation time.

The findings of our study reveal that adding dexamethasone to ropivacaine significantly prolongs the analgesic effect of plain ropivacaine postoperatively (difference of 56 minutes). These findings are consistent with prior research using dexamethasone; however, precise comparisons are difficult due to the range of local anesthetic mixtures and adjuvants used, various blocks evaluated, and different ways of measuring block duration [9].

The present study is among the few that focused on assessing the utility of ropivacaine and ropivacaine with dexamethasone in patients for ultrasound-guided pericapsular nerve group (PENG) block for hip surgery. In our study, we observed a significantly longer (445 minutes) duration of postoperative analgesia, besides a reduced requirement of rescue analgesia dose in patients who received dexamethasone as an adjuvant to ropivacaine. The current study has some limitations, including the non-consideration of analgesics given to the patients in wards before being shifted to the operation room. Further, patient satisfaction in general with anesthesia care should have been assessed 24 hours postoperatively. The small sample size is another limitation.

## Conclusions

Ultrasound-guided PENG block is an effective modality to attenuate perioperative pain while positioning hip fracture patients for performing spinal anesthesia. Dexamethasone, as an adjuvant to ropivacaine, prolongs the duration of analgesia and decreases the requirement for opioids in the postoperative period. Further studies should be conducted with a larger sample size to establish the effectiveness of PENG block for hip surgeries.
